# The Use of Antiseptics in Dentistry

**Published:** 1890-01

**Authors:** 


### The Use of Antiseptics in Dentistry.

It is quite certain that cleanliness and the use of antiseptics in the practice of surgery have come to be regarded as the basis of the successful treatment of wounds, and we take it that what is applicable in this respect to general surgery, is equally to be applied to the special branches of the surgical art. Dental forceps and the various other appliances and instruments which form the armamentarium of the nineteenth century dentist, owing to their particular construction,demand careful and thorough cleansing. A few moment's reflection upon the nature of the work they are designed to accomplish will clearly show how necessary a procedure such cleansing becomes. The mouth, for example, is a vast storehouse for germs, and is the seat of certain diseases, whose propagation by contagion has been repeatedly verified. Special mention may be made in this regard of syphilis and diphtheria. It is quite possible to conceive that the contagion of either of these disorders could be conveyed through the medium of dental forceps whose perfect cleanliness had been inadvertently overlooked. Indeed, judging from some facts which have been brought to our knowledge within the past few days, it would seem that one of the diseases above referred to had been so propagated. Cases of this description, of course, are fortunately supremely difficult of proof, and we presume could only very rarely occur. But the possibility of the occurrence of any such untoward event having been admitted, it would seem to follow as a matter of course that the dental practitioner would exercise a zealous watch over the cleanliness of his instruments, and a reference to Foy's or Hewitt's recent monographs on "The Use of Anæsthetics" would enlighten him on many points. There are so many effectual and facile means of sterilizing the latter, or rendering them antiseptically clean, that the adoption of the particular method of giving effect to the practice is merely a matter of detail. We do not know to what extent dentists use antiseptics in their practice, but assuming for the moment that they do not, we can not see that they would lose in any way if they did. Rather, the principle concerned being a sound one, its enforcement, as we strongly believe, would only be productive of good.—*Med. Press and Circular.*

